# 311 Impact of Speed Buttons and Default Durations on Outpatient Antibiotic Prescribing in Adults

**DOI:** 10.1017/ash.2026.10662

**Published:** 2026-06-23

**Authors:** Diane Yi, Michael Anderson, Shamta Warang, Lynn Reynolds, Sue Milligan, Tatyana Kiryutina, Addis Bedada, Steven Woods, Ryan Paradis, Eleen Daley, Gebre Tiga, Alexis LaCrue, Arunachalam Ramaiah, Karen Williams, JoAnna Wagner

**Affiliations:** 1 Georgia Department of Public Health; 2 Georgia Public Health Laboratory

## Abstract

**Background:** Carbapenemase-producing carbapenem-resistant Acinetobacter baumannii (CP-CRAB) is a highly-transmissible multidrug-resistant organism. In November 2024, the Georgia Department of Public Health (DPH) identified a multi-facility CP-CRAB outbreak in central Georgia and applied whole-genome sequencing (WGS) to guide containment. **Methods:** During November 2024–May 2025, Georgia and Tennessee Public Health Laboratories confirmed 31 CP-CRAB cases. 24 isolates underwent WGS on Illumina MiSeq; sequences were processed with CDC’s PHoeNIx pipeline, and single-nucleotide polymorphism (SNP) heatmaps were generated with Snippy and Python. Epidemiologic data were abstracted from medical records; risk factors and network analyses were conducted using Excel, with case pairs ?10 SNPs prioritized for analysis. **Results:** WGS identified 18 cases in three sub-clusters, plus 2 related and 4 unrelated cases; SNP distances ranged 0–34, with 16/49 (33%) case pairs ?10 SNPs. Facilities included four acute-care hospitals (ACH-1–ACH-4, primary ACH-1), with prior exposures to 3 skilled-nursing facilities (SNFs), 1 ventilated-SNF (vSNF), and 1 long-term ACH (LTACH). Sub-cluster 1 (n = 8; 0–26 SNPs; 12/29 pairs ?10 SNPs) specimen sources included 3 respiratory, 2 urinary, 2 wound, and 1 blood specimens from ACH-1–ACH-4. 6 cases formed 12 ?10-SNP pairs, with collection dates 3–52 days apart; 3/6 cases were admitted to ACH-1 from vSNF with direct overlap, 4/6 had prior exposure to ACH-1, and 2/6 had no epidemiologic links. Prior healthcare exposure and intra-facility (ACH-1) movement were observed among all 6 cases. Sub-cluster 2 (n = 5; 2–22 SNPs; 3/10 ?10) involved 5 ventilated respiratory cases, 4 from ACH-1 and 1 from ACH-2. **Conclusions:** Integrating WGS with epidemiologic data revealed sub-clusters—multiple introductions, distinct transmission pathways, and related cases with wide ranges between collection dates—enabling sub-cluster-specific containment recommendations. These findings informed Georgia’s first multi-facility CP-CRAB outbreak investigation, contributing to the development of a multi-facility engagement model that improved interfacility communication and provider awareness of transmission. Routine WGS–epidemiologic integration is critical for targeted containment and coordinated regional response.

